# Knowledge Distillation-Enhanced Behavior Transformer for Decision-Making of Autonomous Driving

**DOI:** 10.3390/s25010191

**Published:** 2025-01-01

**Authors:** Rui Zhao, Yuze Fan, Yun Li, Dong Zhang, Fei Gao, Zhenhai Gao, Zhengcai Yang

**Affiliations:** 1 College of Automotive Engineering, Jilin University, Changchun 130025, China; rzhao@jlu.edu.cn (R.Z.); fanyz23@mails.jlu.edu.cn (Y.F.); gaozh@jlu.edu.cn (Z.G.); 2 Graduate School of Information and Science Technology, The University of Tokyo, Tokyo 113-8654, Japan; li-yun@g.ecc.u-tokyo.ac.jp; 3 Department of Mechanical and Aerospace Engineering, Brunel University London, Uxbridge UB8 3PH, UK; dong.zhang@brunel.ac.uk; 4 National Key Laboratory of Automotive Chassis Integration and Bionics, Jilin University, Changchun 130025, China; 5 Key Laboratory of Automotive Power Train and Electronics, Hubei University of Automotive Technology, Shiyan 442002, China; yang516516@163.com

**Keywords:** imitation learning, reinforcement learning, behavior transformer, autonomous driving, knowledge distillation, decision-making

## Abstract

Autonomous driving has demonstrated impressive driving capabilities, with behavior decision-making playing a crucial role as a bridge between perception and control. Imitation Learning (IL) and Reinforcement Learning (RL) have introduced innovative approaches to behavior decision-making in autonomous driving, but challenges remain. On one hand, RL’s policy networks often lack sufficient reasoning ability to make optimal decisions in highly complex and stochastic environments. On the other hand, the complexity of these environments leads to low sample efficiency in RL, making it difficult to efficiently learn driving policies. To address these challenges, we propose an innovative Knowledge Distillation-Enhanced Behavior Transformer (KD-BeT) framework. Building on the successful application of Transformers in large language models, we introduce the Behavior Transformer as the policy network in RL, using observation–action history as input for sequential decision-making, thereby leveraging the Transformer’s contextual reasoning capabilities. Using a teacher–student paradigm, we first train a small-capacity teacher model quickly and accurately through IL, then apply knowledge distillation to accelerate RL’s training efficiency and performance. Simulation results demonstrate that KD-BeT maintains fast convergence and high asymptotic performance during training. In the CARLA NoCrash benchmark tests, KD-BeT outperforms other state-of-the-art methods in terms of traffic efficiency and driving safety, offering a novel solution for addressing real-world autonomous driving tasks.

## 1. Introduction

Autonomous driving technology has seen increasingly widespread applications in the transportation sector, becoming a key force driving the development of intelligent transportation systems [1,2,3,4]. This technology holds the promise of significantly improving traffic efficiency and reducing accident risks caused by human errors, thereby impacting future mobility in profound ways. Autonomous driving systems are typically composed of several core modules, including perception, decision-making, and motion control. Among these, decision-making plays a crucial role as the bridge between perception and control [5,6,7,8,9,10,11,12]. The decision-making system is responsible for autonomous judgment and choice-making within complex traffic environments, covering areas such as path planning, obstacle avoidance, lane-keeping, and interactions with other traffic participants. Accurate and efficient decision-making is critical to ensuring the safety, reliability, and user experience of autonomous driving systems.

Traditional approaches to decision-making are generally divided into rule-based, optimization-driven, and utility-based strategies. Common rule-based methods include standard-based approaches [13], finite state machines [14], and Bayesian networks [15], among others. While these methods are straightforward to implement, their application scope is limited. Typical optimization-based methods, such as techniques for decision generation [16] or goal trajectory planning [17], can often find optimal solutions but face challenges when dealing with model-free scenarios. Utility function-based methods have simple structures and usually take into account metrics such as safety, comfort, efficiency, and traffic rules [18]. However, selecting and balancing these metrics can be complex. In motion planning, there are two main frameworks. The first framework involves using geometric curves [19,20,21] for trajectory planning, followed by trajectory tracking based on proportional–integral–derivative (PID) control [22], sliding mode control (SMC) [23], or model predictive control (MPC) [24]. The second framework treats planning and tracking as a unified whole, handling the coupling relationship between the two [25]. Research in this area is already well established, especially with MPC-based methods [26,27], which excel in handling multiple constraints and are easily integrated with traffic prediction [28]. However, traditional decision-making methods lack generalization capabilities and robustness, making them less adaptable to complex and stochastic environments.

To achieve advanced autonomous driving in dynamic and open environments, Imitation Learning (IL) and Reinforcement Learning (RL) techniques have been widely studied. IL learns from expert demonstration datasets, with the most common approach being Behavioral Cloning (BC). For example, methods based on Support Vector Machines (SVMs) [29] and Long Short-Term Memory (LSTM) networks [30] have been extensively applied to decision-making [31]. To apply IL in multi-task learning, Codevilla et al. [32,33] proposed Conditional Imitation Learning (CIL) to learn both low-level controls and high-level instructions from human drivers. Xiao et al. [34] further utilized CIL to address multimodal issues. Chen et al. [35] introduced the LBC method, using privileged information in simulators to train IL models. Ozcelik et al. [8] proposed a method combining Generative Adversarial Imitation Learning (GAIL) with Curriculum Learning (CL) to imitate expert driving behavior on highways. Tian et al. [9] introduced an IL method that enabled a model predictive control (MPC)-based lane-changing strategy through limited demonstration learning. Additionally, to address domain transfer issues, Li et al. [36] developed an off-policy IL method based on knowledge distillation. To enhance IL stability and interpretability, Teng et al. [37] proposed a two-stage IL framework incorporating bird’s-eye-view (BEV) masks, and Wang et al. [38] introduced a BC approach that extracts explicit features from real-world trajectories. Another typical IL method is Inverse Learning, which derives parametric planning objectives by learning from human demonstrations [39].

However, despite the advantages of IL in terms of high sample efficiency and rapid learning, it still faces challenges related to distributional shift. RL, which updates policies through interaction with the environment, shows great potential in mitigating these inherent IL issues. Hoel et al. [6] proposed a policy based on Deep Q-Networks (DQNs) to determine lane selection and acceleration decisions for autonomous vehicles. Tang et al. [7] introduced a decision-making strategy based on Soft Actor–Critic (SAC), which performed well in highway driving scenarios. Fu et al. [5] presented a decision framework based on Deep Deterministic Policy Gradient (DDPG) for emergency maneuver control, demonstrating its effectiveness in handling critical situations. Kamran et al. [10] combined DQN with MPC for decision-making, impacting the low-level planner in merging scenarios. Valiente et al. [11] utilized DQN for navigation in environments such as highways and roundabouts. Zhang et al. [12] developed a two-tier lane-changing system that combines rule-based and RL methods to enhance vehicle cooperation. To address uncertainty in autonomous driving, researchers proposed robust Actor–Critic methods [40,41] to cope with traffic uncertainties and avoid complex motion dynamics modeling. Wu et al. [42] built an action-conditioned ensemble model to evaluate environmental uncertainty and combined it with model-based RL. Additionally, to improve computational efficiency, Li et al. [43] developed a lightweight Transformer model for image semantic extraction and integrated it with RL methods. Chen et al. [44] proposed WOR, assuming “world on rails” to enable model-based RL training. Zhao et al. [45] introduced CADRE, which first trains a Co-attention Perception Module (CoPM), then freezes this module and trains it with Proximal Policy Optimization (PPO). Chekroun et al. [46] proposed GRIAD, which processes both offline datasets and data explored via online RL. Coelho et al. [47] introduced RLfOLD, incorporating a policy network outputting two standard deviations to help the agent adapt to the inherent uncertainty levels of IL and RL.

Despite the substantial potential of RL in decision-making, its policy networks still lack sufficient inference capabilities for complex driving environments. Transformer models [48], which have demonstrated strong reasoning abilities in natural language processing and computer vision, show promise for solving complex decision-making problems in autonomous driving. Recent studies have successfully applied Transformers to various autonomous driving tasks. Chitta et al. [49] used a multimodal Transformer to fuse camera and LiDAR data for better environmental perception. Shao et al. [50,51] utilized Transformers for temporal behavior inference and hazard prediction. Wang et al. [52] and Xu et al. [53] developed Transformer-based systems for driving decisions and behavior explanation. However, applying Transformers to RL in autonomous driving faces challenges. RL methods suffer from low sample efficiency, requiring millions of interaction steps for training. Research by Toromanoff et al. [54] shows that even with pretraining, convergence needs over 20 million steps. Additionally, Transformer models’ complexity increases computational demands and training difficulty.

To address these issues, this study proposes a novel Knowledge Distillation-Enhanced Behavior Transformer (KD-BeT), aimed at improving RL efficiency and performance in autonomous driving decision-making. Specifically, we used IL to train a teacher model quickly and accurately, enabling it to make optimal decisions in complex scenarios. Subsequently, during the RL training process, we modeled the autonomous driving decision problem as a Partially Observable Markov Decision Process (POMDP) and leveraged knowledge distillation to transfer the teacher model’s knowledge to the Transformer student model, thus enhancing the training efficiency of the student model. We employed PPO as the policy update algorithm. During training, we gradually decayed the teacher model’s influence, initially relying more on its guidance to expedite convergence, and later, as the teacher model’s influence diminishes, the Transformer student model increasingly utilized its own capabilities, ensuring high final performance. The knowledge distillation mechanism enables efficient knowledge transfer from a compact teacher model to the student model, significantly reducing the required training samples while maintaining high performance. The Transformer architecture, with its powerful attention mechanism and sequential processing capabilities, excels at capturing temporal dependencies and contextual relationships in driving scenarios, leading to more informed decision-making. This approach not only addresses the traditional sample inefficiency issue of RL but also significantly enhances decision accuracy and generalization capabilities in complex driving scenarios.

The main contributions of this study include the following:

- Propose the Behavior Transformer as a novel policy network for autonomous driving decision-making in RL, which innovatively processes temporal sequences of historical observations and actions as input. By harnessing the Transformer’s powerful contextual learning capabilities and attention mechanisms, this approach significantly enhances both decision-making accuracy and generalization ability.
- Develop an innovative knowledge distillation framework that establishes a teacher–student paradigm to boost RL training efficiency. The teacher model swiftly and precisely acquires expert knowledge through IL, while the student model accelerates learning through knowledge transfer. Furthermore, an adaptive decaying coefficient gradually reduces the teacher’s influence, enabling the student model to fully develop its capabilities and ultimately surpass the teacher’s performance.
- Perform comprehensive evaluations on the CARLA NoCrash benchmark suite, with extensive experimental results demonstrating that KD-BeT achieves state-of-the-art performance in terms of both decision-making accuracy and generalization capabilities across various challenging driving conditions.

The rest of this paper is organized as follows: Section 2 introduces the framework of the autonomous driving method based on KD-BeT. In Section 3, the decision-making problem in autonomous driving is formulated as a POMDP problem, including a description of the observation space, action space, and reward function. Section 4 provides a detailed explanation of the proposed KD-BeT method, designed to solve the aforementioned POMDP problem. Section 5 presents the experimental study, where the performance of KD-BeT is evaluated through benchmark tests. Finally, Section 6 concludes the paper.

## 2. Method Framework for Decision-Making of Autonomous Driving Based on KD-BeT

**Figure 1:**
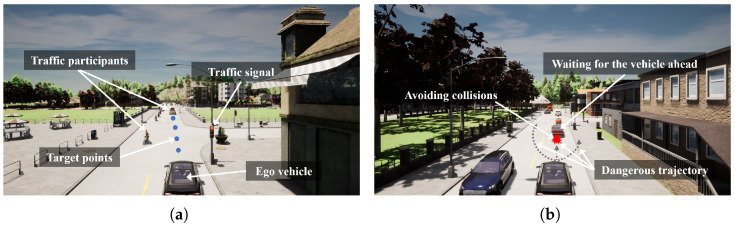
Problem definition illustration: (**a**) The ego vehicle must comply with traffic rules to reach its destination safely and efficiently. (**b**) The ego vehicle must avoid obstacles and prevent lane departure to ensure safety.

In this study, the driving goal for the ego vehicle is to safely and efficiently travel from the starting point to the destination while adhering to traffic rules. The task scenario involves a dynamic traffic environment, including other vehicles, traffic participants, traffic signals, and varying road conditions. The autonomous driving system must handle navigation tasks, follow traffic regulations, and perceive and avoid obstacles in a dynamic environment to ensure driving safety, as illustrated in Figure 1. Additionally, the ego vehicle needs to complete the route efficiently within the allotted time, avoiding unnecessary delays and congestion.

The problem is defined as follows: At each time step in RL, the ego vehicle receives observation information, including its own motion state, information about surrounding traffic participants, traffic rules (e.g., traffic lights), and the given target point. The ego vehicle, combining historical observation and action sequences, uses the policy network to decide the current time step’s acceleration and steering angle. If the vehicle ahead slows down or changes lanes, the ego vehicle must take timely actions, such as avoiding or slowing down, to ensure driving safety. Meanwhile, the ego vehicle should maintain a reasonable speed to avoid unnecessary delays and ensure efficient task completion. When encountering traffic lights or other traffic rules, the ego vehicle must strictly comply to ensure safe driving.

**Figure 2:**
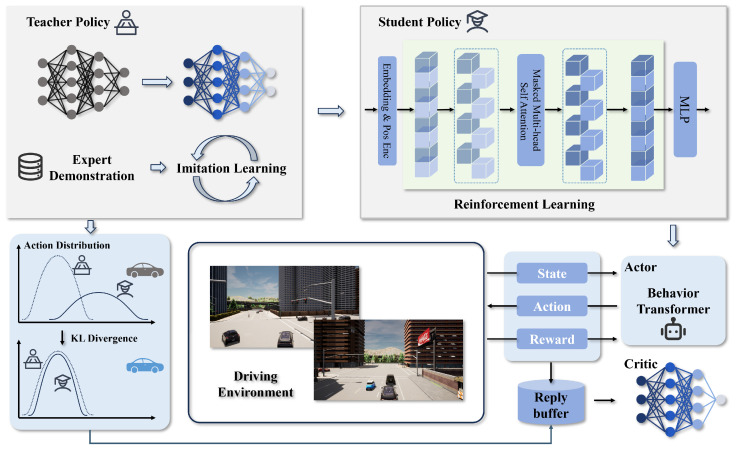
The proposed KD-BeT framework consists of two main components: a teacher policy trained using IL and a student policy trained using RL. The teacher policy accelerates the training efficiency of the student policy through knowledge distillation during the RL process.

As shown in Figure 2, the framework of this study is divided into two main stages: teacher policy training and student policy training. The teacher policy is trained through IL. First, an expert agent collects a dataset as a demonstration, and a small-capacity teacher model is trained quickly and accurately using the BC method. Through this process, the teacher model learns expert behaviors from the demonstration dataset, laying a solid foundation for the subsequent RL phase.

After completing the teacher policy training, the process moves to the student policy training stage. In this stage, the Behavior Transformer model is proposed as the student model and is trained within the RL framework. Knowledge distillation is employed to effectively transfer the knowledge learned by the teacher model to the student model, thereby improving the training efficiency of the student model. As training progresses, the influence of the teacher policy gradually decreases, enabling the student model to exceed the teacher’s performance, fully realize its potential, and achieve greater accuracy and efficiency in executing decisions.

This two-stage training framework, combining IL and RL with knowledge distillation, leverages expert-level decision-making for fast convergence while enabling the student model to surpass initial limitations. Through a combination of direct imitation and gradual reinforcement, the Behavior Transformer model can generalize more effectively, ensuring robust performance in dynamic environments.

## 3. Partially Observable Markov Decision Process for Decision-Making of Autonomous Driving

To address the autonomous driving decision-making problem described above, this paper adopts a Markov modeling approach, formalizing the autonomous driving decision problem as a POMDP. This section first introduces the basic concepts of POMDP, then provides a detailed description of the observation and action representations of the ego vehicle, and finally explains the reward function design.

### 3.1. Partially Observable Markov Decision Process

The POMDP framework allows an agent to reason and make decisions when the state is not fully observable. By introducing an observation space and an observation function, the agent can make decisions based on noisy observation information. The components of a POMDP are as follows:

- S: State space, representing the set of all possible system states.
- A: Action space, representing the set of all possible actions the agent can take.
- $T(s,a,s^{\prime })$: State transition probability, representing the probability of transitioning from state *s* to state $s^{\prime }$ after taking action *a*.
- R(s,a): Reward function, representing the reward received when taking action *a* in state *s*.
- O: Observation space, representing the set of possible observations the system can receive.
- $Z(o,s^{\prime },a)$: Observation probability, representing the probability of observing *o* after taking action *a* and transitioning to a new state $s^{\prime }$.
- γ: Discount factor, used to discount the influence of future rewards.

Under the POMDP framework, the ego vehicle infers the current hidden state from the received partial observation information and reasons to maximize the future cumulative reward. This enables the vehicle to make decisions in complex traffic environments, such as timely avoiding obstacles or other vehicles while maintaining lane stability. When faced with changing road and traffic conditions, POMDP allows the vehicle to take actions based on uncertain environmental information, thereby enhancing the safety and robustness of autonomous driving.

### 3.2. Observation Representation

In autonomous driving decision-making research, a well-designed observation space is critical for RL training, as it directly determines the environmental information that the ego vehicle can perceive and understand. In this study, the observation space of the ego vehicle consists of three main components: the ego vehicle’s current motion state, information about surrounding traffic participants, and information about the given target point. These elements together provide comprehensive perceptual input for the ego vehicle’s driving decisions, enabling it to handle complex and dynamically changing traffic environments. The formal representation of the observation space is as follows:

(1) $$O=O_{\mathrm{Ego}}\cup O_{\mathrm{Traffic}}\cup O_{\mathrm{Target}}$$

where $O_{\mathrm{Ego}}$ represents the ego vehicle’s motion state, covering information such as the current speed, position, and heading. $O_{\mathrm{Traffic}}$ provides detailed data about surrounding traffic participants, including their relative position, speed, and traffic signal status. $O_{\mathrm{Target}}$ contains the relative position of the target point, which is used to guide the ego vehicle’s path planning and navigation. The ego vehicle’s motion state $O_{\mathrm{Ego}}$ is defined in detail as follows:

(2) $$O_{\mathrm{Ego}}=\left\{v_{\mathrm{ego}},v_{\mathrm{ref}},d_{\mathrm{lat}},d_{\mathrm{lon}},\Delta \psi \right\}$$

where $v_{\mathrm{ego}}$ represents the real-time speed of the ego vehicle, reflecting its dynamic characteristics at the present moment. $v_{\mathrm{ref}}$ denotes the target speed of the ego vehicle, typically set by the control strategy to ensure smooth and efficient driving. $d_{\mathrm{lat}}$ and $d_{\mathrm{lon}}$ represent the lateral and longitudinal distances between the ego vehicle and the next target point, which are crucial for lane-keeping, obstacle avoidance, and future path planning. Δψ is the heading angle difference between the ego vehicle and the target point, ensuring that the vehicle can steer accurately and effectively track the planned path. The information about surrounding traffic participants $O_{\mathrm{Traffic}}$ includes

(3) $$O_{\mathrm{Traffic}}=\left\{\sigma_{\mathrm{sig}},\xi_{\mathrm{part}}\right\}$$

where $\sigma_{\mathrm{sig}}$ represents the status of traffic signals, such as red, green, or yellow lights. These signals directly affect the driving decisions of the ego vehicle, especially in complex scenarios like intersections. $\xi_{\mathrm{part}}$ represents the relative information of surrounding traffic participants, including their position, speed, and heading angle relative to the ego vehicle. By continuously monitoring this information, the ego vehicle can respond in a timely manner to avoid collisions and maintain a safe distance. Finally, the target point information $O_{\mathrm{Target}}$ is defined as

(4) $$O_{\mathrm{Target}}={\left\{\left(\Delta x_{i},\Delta y_{i}\right)\right\}}_{i=1}^{N}$$

where $\left(\Delta x_{i},\Delta y_{i}\right)$ represent the lateral and longitudinal distances of the *i*-th target point relative to the ego vehicle’s coordinate system. *N* is the number of target points, serving as a core input for path planning. By continuously tracking these target points, the ego vehicle can ensure that its path planning adapts to the current traffic and road conditions, achieving smooth and safe driving. Additionally, these target points provide the ego vehicle with clear navigational direction, helping it accurately reach its intended destination.

By integrating these three components, the observation space provides the ego vehicle with comprehensive and detailed environmental information, enabling it to make informed decisions in complex traffic environments. This design not only enhances the vehicle’s perception of its surroundings but also improves the safety and efficiency of its driving behavior.

### 3.3. Action Representation

In autonomous driving decision-making research, the design of the action space is as critical as that of the observation space, as it directly influences the effectiveness of the vehicle’s interaction with the environment. Previous studies have primarily employed two common types of action representations: trajectory waypoints and control signals. The trajectory waypoint method guides vehicle motion through predefined paths. However, Wu et al. [55] demonstrated that this method has limitations in certain operational scenarios. For example, during sharp turns or starting from a stationary position at traffic lights, the accuracy of the predefined trajectory may decline, leading to unstable control. Additionally, the trajectory waypoints approach requires the use of additional PID controllers to convert the planned trajectory into actual throttle, brake, and steering control signals. This not only increases the system’s complexity but also requires fine-tuning of the PID parameters, which may negatively impact the system’s overall performance. In contrast, the control signal output directly provides the necessary commands to the vehicle’s actuators, simplifying the control system and avoiding the intermediate trajectory conversion step. Although this method is focused on the current time step, it avoids the complexities associated with trajectory conversion. However, relying solely on control signals for the current time step may result in delayed responses to potential future hazards, as the vehicle lacks foresight of upcoming events.

To address this limitation, this study employs a Transformer model that combines state and action sequences with contextual information to predict future control signals. The self-attention mechanism of the Transformer allows the model to capture long-range dependencies and potential future threats from the global temporal context, reducing response delays and enhancing adaptability in complex traffic scenarios. Based on these considerations, we opted to directly output control signals as the action space to reduce system complexity and improve control response efficiency. Specifically, the action space A is defined as

(5) $$A=\{a_{\mathrm{acc}},\delta_{\mathrm{steer}}\}$$

where $a_{\mathrm{acc}}$ represents the acceleration control command, which influences the vehicle’s speed through throttle and brake inputs, and $\delta_{\mathrm{steer}}$ represents the steering angle control command, determining the vehicle’s directional changes. To effectively model these control signals, this study employs a Beta distribution, denoted as Beta(α,β), where α and β are shape parameters that control the concentration of the distribution around 0 and 1, respectively. The probability density function of the Beta distribution is defined as

(6) $$f\left(x;\alpha ,\beta \right)=\frac{\Gamma (\alpha +\beta )}{\Gamma (\alpha )\Gamma (\beta )}\;x^{\alpha -1}{\left(1-x\right)}^{\beta -1},\;x\in \left[0,1\right],\;\alpha >0,\;\beta >0$$

where Γ(·) is the Gamma function. Choosing the Beta distribution has several advantages. First, the Beta distribution is defined on the interval [0,1], which naturally constrains control signals within a valid range without requiring additional activation functions to enforce limits. This aligns well with the physical constraints of vehicle control signals, such as the range of acceleration and steering angle. Secondly, by adjusting the parameters α and β, the Beta distribution can take on various shapes, including uniform, unimodal, or bimodal distributions. This flexibility allows it to capture a wide range of control behaviors in driving scenarios, such as smooth acceleration or emergency braking. Additionally, the Beta distribution can represent multimodal distributions, which is especially useful in driving contexts, as the vehicle may need to perform extreme control actions (e.g., emergency braking or sharp turns).

### 3.4. Reward Function

In autonomous driving, the vehicle’s objective is to efficiently complete navigation tasks while ensuring safe and smooth driving. To achieve this goal, the reward function designed in this study consists of multiple components, each optimized for specific driving requirements. These components include a speed reward, position reward, orientation reward, action reward, and termination reward. The weights of these components are represented by $\omega_{i}$ and $C_{i}$, ensuring a proper balance across different objectives. By aggregating these sub-reward functions, the overall reward function effectively reflects safety, efficiency, and driving comfort. It is defined as follows:

(7) $$r=r_{\mathrm{speed}}+r_{\mathrm{position}}+r_{\mathrm{rotation}}+r_{\mathrm{steer}}+r_{\mathrm{terminal}}$$

First, the speed reward $r_{\mathrm{speed}}$ measures the difference between the current speed of the ego vehicle and the desired speed, aiming to encourage the ego vehicle to drive close to the target speed. It is defined as follows:

(8) $$r_{\mathrm{speed}}=\omega_{1}\cdot \left(1-\frac{|v_{\mathrm{ego}}-v_{\mathrm{desired}}|}{v_{\max}}\right)$$

where $v_{\mathrm{ego}}$ is the current speed of the ego vehicle, $v_{\mathrm{desired}}$ is the target speed, and $v_{\max}$ is the maximum speed. This term incentivizes the ego vehicle to optimize its speed performance while ensuring smooth driving.

Next, the position reward $r_{\mathrm{position}}$ is calculated based on the lateral deviation of the ego vehicle from the centerline of the target route. By penalizing excessive deviation, this term encourages the ego vehicle to stay on the predefined trajectory, defined as follows:

(9) $$r_{\mathrm{position}}=-\omega_{2}\cdot \Delta_{\mathrm{lateral}}$$

where $\Delta_{\mathrm{lateral}}$ represents the lateral distance between the center of the ego vehicle and the centerline of the route. Maintaining accurate lane positioning is essential for safe driving.

The rotation reward $r_{\mathrm{rotation}}$ helps the ego vehicle maintain the correct driving direction by assessing the angular difference between the vehicle’s heading and the route’s heading. It is defined as

(10) $$r_{\mathrm{rotation}}=-\omega_{3}\cdot \Delta_{\mathrm{angular}}$$

where $\Delta_{\mathrm{angular}}$ is the absolute angular difference between the ego vehicle’s heading and the route heading. This reward term enhances the ego vehicle’s path-following capability by reducing heading error.

The steer reward $r_{\mathrm{steer}}$ is used to limit abrupt changes in steering, avoiding excessive fluctuations in steering angle between consecutive time steps. It is defined as

(11) $$r_{\mathrm{steer}}=\left\{\begin{array}{cc} -C_{1} & \mathrm{if}\;|\delta_{\mathrm{current}}-\delta_{\mathrm{previous}}|\;>0.01\\ 0 & \mathrm{otherwise} \end{array}\right.$$

where $\delta_{\mathrm{current}}$ and $\delta_{\mathrm{previous}}$ represent the steering angles at the current and previous time steps, respectively. This term ensures smooth steering adjustments, enhancing driving comfort and stability.

Finally, the terminal reward $r_{\mathrm{terminal}}$ applies in cases where the episode terminates, typically triggered by unsafe behaviors such as running a red light, collisions, or veering off-route. It is defined as

(12) $$r_{\mathrm{terminal}}=\omega_{4}\cdot \left(-1-v_{\mathrm{ego}}\right)$$

This reward imposes a significant penalty when unsafe events occur, and applies more severe penalties at higher speeds to reflect the greater negative impact in high-risk scenarios.

The reward function design of the KD-BeT framework considers multiple key dimensions in autonomous driving decision-making. The speed reward $r_{\mathrm{speed}}$ guides the vehicle to maintain appropriate speed by evaluating the difference between actual and desired speeds. The position reward $r_{\mathrm{position}}$ ensures correct path-following by calculating lateral deviation from the lane center. The rotation reward $r_{\mathrm{rotation}}$ helps maintain proper heading direction by measuring angular difference. These three rewards together evaluate driving efficiency. For comfort, the steering reward $r_{\mathrm{steer}}$ limits abrupt steering changes between consecutive time steps by applying penalty $C_{1}$ when steering angle change exceeds 0.01. For safety, the terminal reward $r_{\mathrm{terminal}}$ penalizes dangerous behaviors like running red lights or collisions, with higher penalties at higher speeds reflecting increased risk. The weights $\omega_{1}$ to $\omega_{4}$ and $C_{1}$ enable flexible adjustment between multiple objectives. The immediate rewards (speed, position, rotation, steering) provide short-term feedback while the terminal reward evaluates long-term safety. This multi-level reward mechanism enables balanced decision-making considering safety, efficiency, and comfort across complex scenarios.

## 4. Knowledge Distillation-Enhanced Behavior Transformer for Decision-Making of Autonomous Driving

Based on the proposed POMDP problem, this section introduces how the KD-BeT model addresses this issue. First, we present the Behavior Transformer for sequential decision-making, followed by a detailed description of the training processes for the teacher and student models, and finally, an explanation of the entire algorithm’s implementation workflow.

### 4.1. Behavior Transformer for Sequential Decision Making

In autonomous driving, effectively utilizing contextual temporal information is crucial for accurate and efficient decision-making. The current decisions in driving environments depend not only on immediate observations but also on historical behaviors and environmental changes. Therefore, temporal information plays a vital role in the decision-making process, helping models capture temporal dependencies and better understand the dynamic changes in driving scenarios. To address this challenge, sequence modeling has been widely applied to decision-making tasks, enabling the extraction of potential temporal dependencies from historical observation–action pair sequences to inform current decisions.

**Figure 3:**
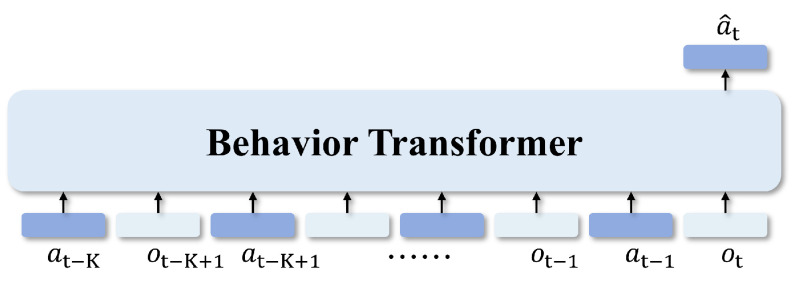
The student policy, based on the Behavior Transformer, utilizes contextual temporal information as input to predict the current action based on the observation and action from previous time steps.

In this context, the Transformer model offers significant advantages in handling sequential decision-making tasks. Through its self-attention mechanism, it captures long-range dependencies while avoiding gradient vanishing or exploding problems that Recurrent Neural Networks (RNNs) might encounter in long sequences. The student policy employs a Transformer model for sequential decision-making, utilizing contextual temporal information to predict the next action. To fully leverage this temporal information, the student policy uses a context window of length *K*, processing historical observation–action pairs from time t−K to *t*, including $o_{t}$, $a_{t-1}$, $o_{t-1}$, *…*, $o_{t-K+1}$, $a_{t-K}$, to predict the next action ${\hat{a}}_{t}$, as shown in Figure 3. In the Transformer, each token represents an observation–action pair, containing current observation information and corresponding action decisions. As sequential input units, tokens carry environmental states and decision behaviors at each time step, helping the model to capture temporal dependencies. A detailed introduction to the Transformer can be found in Appendix A.

**Figure 4:**
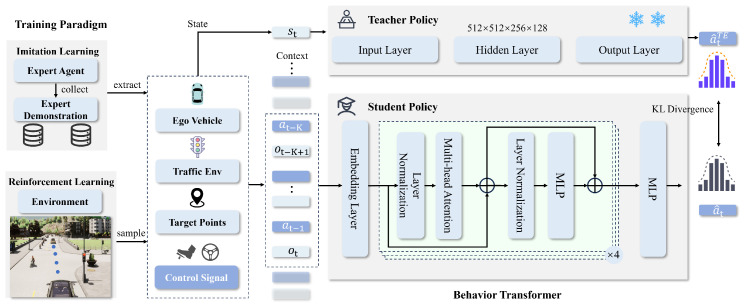
Illustration of the knowledge distillation process, where an MLP-based teacher policy learns from expert demonstrations through Imitation Learning while a Behavior Transformer-based student policy leverages contextual data from environmental interactions through Reinforcement Learning.

Figure 4 illustrates the detailed architecture of KD-BeT. As shown in the figure, the KD-BeT architecture primarily consists of a teacher model and a student model. The teacher model, based on multi-layer perceptron (MLP), learns driving strategies from expert demonstration data and transfers these strategies to the student model through knowledge distillation. The student model, based on the Behavior Transformer, utilizes contextual data from environmental interactions for sequential decision-making and is trained through Reinforcement Learning. The discrepancy between the teacher and student model outputs is measured using KL divergence. Through this approach, the student model can effectively absorb knowledge from the teacher model, enabling efficient decision-making in a shorter time frame.

### 4.2. Knowledge Distillation-Enhanced Behavior Transformer

#### 4.2.1. Imitation Learning for Teacher Model

This study first employs IL to train a smaller-capacity teacher model. This teacher model offers the advantages of making quick decisions, achieving high accuracy, and being easier to train. The teacher model learns driving strategies from expert demonstration data and transfers these strategies to the student model through knowledge distillation. During the IL phase, the teacher model focuses on minimizing the discrepancy between its predictions and the expert demonstration data, ensuring that it can provide effective guidance to the student model in the subsequent RL phase.

In this study, we choose Roach [56] as the expert agent for data collection. Roach adopts a two-stage training approach: first by training expert policy through Reinforcement Learning, then using this policy to collect data for training IL agents. During training, Roach fully utilizes privileged information, including detailed information about roads, lanes, routes, vehicles, pedestrians, traffic signals, and stop signs, and renders this information into bird’s-eye-view images. Compared to traditional rule-based expert systems, this learning-based expert can better capture and transmit rich information beyond direct supervision signals. This capability enables it to extract more subtle driving behavior features, thereby significantly improving the training effectiveness of downstream models, ultimately achieving more intelligent decision-making in complex driving scenarios.

During the IL phase, the primary objective of the teacher model is to learn and approximate the optimal policy by minimizing the error between its outputs and the expert demonstration data. The core of this process lies in optimizing the parameters of the teacher model to ensure that the actions it outputs under various observation conditions closely match those in the expert demonstration data. To achieve this, the Mean-Squared Error (MSE) was chosen as the loss function in this study. The specific form of the MSE loss function is

(13) $$L^{\mathrm{TE}}\left(\pi_{\theta }^{\mathrm{TE}}\right)=E\left[{∥a_{t}^{E}-{\hat{a}}_{t}\left(\pi_{\theta }^{\mathrm{TE}}\right)∥}^{2}\right]$$

where $a_{t}^{E}$ represents the expert action at time step *t* in the expert demonstration data, ${\hat{a}}_{t}$ is the action predicted by the teacher model under the state $s_{t}$, and $\pi_{\theta }^{\mathrm{TE}}$ denotes teacher policy.

Based on the aforementioned loss function, the parameters of the teacher model are optimized iteratively. Specifically, in each iteration, the model parameters are updated according to the following formula:

(14) $$\theta_{k+1}^{\mathrm{TE}}\leftarrow \theta_{k}^{\mathrm{TE}}-\alpha_{IL}\cdot \nabla L^{\mathrm{TE}}\left(\pi_{\theta }^{\mathrm{TE}}\right)$$

where $\alpha_{IL}$ represents the learning rate in IL process and ∇ denotes the gradient of the loss function $L^{\mathrm{TE}}\left(\pi_{\theta }^{\mathrm{TE}}\right)$ with respect to the parameters θ.

Through the aforementioned optimization process, the teacher model can gradually adjust its parameters, enabling it to effectively learn and approximate reasonable driving strategies during the IL phase. This process not only ensures the accuracy of the model when handling expert demonstration data but also lays a solid foundation for further optimization and knowledge transfer through RL.

#### 4.2.2. Reinforcement Learning for Student Model with Knowledge Distillation

After training the teacher model, knowledge distillation is employed to transfer the knowledge learned by the teacher model to the student model. This approach further enhances the performance of the student model, particularly in complex tasks, where it effectively guides the student model in learning the correct policy more rapidly. By incorporating the knowledge of the teacher model into the student model’s learning process, the student model can benefit from the teacher model’s experience, thereby reducing training time and improving the overall quality of the policy. In RL training, knowledge distillation not only helps the student model minimize unnecessary exploration but also enables it to converge more quickly to a higher-performing policy. During this process, we integrated knowledge distillation with PPO to maximize the expected cumulative reward. The student model’s parameters were optimized through the following gradient update formula:

(15) $$\theta_{k+1}=\arg\max_{\theta }E_{\tau ∼\pi_{\theta_{k}}}\left[L_{\mathrm{ppo}}+L_{\mathrm{ent}}+L_{\exp}+L_{\mathrm{imi}}\right]$$

where $L_{\mathrm{ppo}}$ is the core policy gradient objective, which ensures the stability and effectiveness of the model policy through PPO. The PPO algorithm uses a clipping mechanism to prevent excessive changes in policy during updates, thus avoiding instability in model training. The specific PPO objective function is as follows:

(16) $$L_{\mathrm{ppo}}=E_{t}\left[\min\left(r_{t}\left(\theta \right){\hat{A}}_{t},\mathrm{clip}\left(r_{t}\left(\theta \right),1-\epsilon ,1+\epsilon \right){\hat{A}}_{t}\right)\right]$$

where $r_{t}\left(\theta \right)$ is the probability ratio between the new policy $\pi_{\theta }$ and the old policy $\pi_{\theta_{\mathrm{old}}}$, ϵ is the clipping threshold, and ${\hat{A}}_{t}$ is the advantage function. The PPO algorithm is optimized using Generalized Advantage Estimation (GAE) to enhance the model’s estimation of policy advantage, thereby balancing exploration and exploitation in longer-sequence tasks. To further encourage policy exploration by the student model, this study introduces an entropy objective $L_{\mathrm{ent}}$, defined as

(17) $$L_{\mathrm{ent}}=-\lambda_{\mathrm{ent}}\cdot H\left(\pi_{\theta }\left(\cdot |{\left\{o_{t},a_{t}\right\}}_{T_{obs}-K}^{T_{obs}-1}\cup \left\{o_{T_{obs}}\right\}\right)\right)$$

where $\lambda_{\mathrm{ent}}$ is the weight coefficient of the entropy term, $T_{obs}$ denotes the current time step, and H represents the entropy of the policy. By maximizing the entropy of the policy, this entropy objective allows the student model to retain a degree of randomness in action selection, preventing it from prematurely converging to a local optimum. Maximizing entropy encourages the student model to explore a wider range of possibilities, helping it find a more comprehensive policy in complex environments. In addition, this study introduces an exploration objective $L_{\exp}$, which primarily guides the student model to follow predefined rules in specific situations. The formula is as follows:

(18) $$L_{\exp}=\lambda_{\exp}\cdot KL\left(\pi_{\theta }\left(\cdot |{\left\{o_{t},a_{t}\right\}}_{T_{obs}-K}^{T_{obs}-1}\cup \left\{o_{T_{obs}}\right\}\right)\|\pi_{B}\right)$$

where $\lambda_{\exp}$ represents a variable weight coefficient of the exploration term, which activates only in the time steps immediately following termination events. The KL divergence is used to measure the discrepancy between the current policy distribution and the target distribution. $\pi_{B}$ is the distribution associated with specific traffic rules, designed to constrain the model’s actions after termination events occur. Termination events include collisions, running red lights, veering off-route, and blocking. Specifically, when a collision or red light violation occurs, we apply $\pi_{B}=B\left(1,2.5\right)$ to the acceleration to encourage the model to decelerate without altering steering behavior. When the vehicle is blocked, we use the acceleration prior B(2.5,1) to address the situation; if the vehicle deviates from the route, a uniform prior B(1,1) is applied to steering. While this mechanism is somewhat similar to maximizing entropy in certain cases, it optimizes model behavior more effectively under termination conditions by incorporating specific exploration priors, enabling the model to adhere to traffic rules and avoid repeated mistakes. By introducing a combination of indicator functions and exploration priors, this approach better guides the model in correcting erroneous behaviors after termination events. Additionally, $L_{\mathrm{imi}}$ represents the KL divergence objective between the student and teacher models, guiding the student model to learn from the teacher’s policy. It is defined as follows:

(19) $$L_{\mathrm{imi}}=-\lambda_{\mathrm{imi}}\cdot KL\left(\pi_{\theta }\left(\cdot |{\left\{o_{t},a_{t}\right\}}_{T_{obs}-K}^{T_{obs}-1}\cup \left\{o_{T_{obs}}\right\}\right)\|\pi_{\theta }^{\mathrm{TE}}\left(\cdot |o_{T_{obs}}\right)\right)$$

where $\lambda_{\mathrm{imi}}$ represents the weight coefficient of the teacher policy term, which decays linearly throughout the training process. During knowledge distillation, the choice of weight decay strategy significantly impacts model training effectiveness, with common decay strategies including linear decay, exponential decay, cosine decay, and step decay. This study chooses the linear decay strategy because it is not only simple and intuitive to implement, but also provides a smooth and predictable decay process that avoids sudden changes. This strategy maintains strong teacher guidance in the early stages of training to help the student model quickly grasp fundamental knowledge, while gradually reducing teacher influence in later stages to give the student model more opportunities to explore and optimize its own strategy, thereby achieving a natural transition from teacher dependence to independent learning. The linear decay strategy is expressed as

(20) $$\lambda_{\mathrm{imi}}=\lambda_{\mathrm{imi}}^{0}\cdot \left(1-\frac{e}{N_{e}}\right)$$

where $\lambda_{\mathrm{imi}}^{0}$ is an adjustable initial weight coefficient (typically 1.0) used to control the initial intensity of knowledge distillation, *e* represents the current iteration number, and $N_{e}$ represents the total number of iterations. This decay strategy, by setting an appropriate initial weight $\lambda_{0}$, amplifies the knowledge distillation effect of the teacher model in the early stages, allowing the student model to fully utilize the teacher’s guidance, while encouraging the student model to develop its own performance potential in the later stages. KL divergence is used to measure the difference between student model and teacher model policies. By minimizing the KL divergence between student and teacher model policies, this objective helps the student model gradually approach the optimal policy learned by the teacher model, thereby accelerating the training process and improving policy performance. The teacher model’s knowledge serves as a reference for the student model, playing an important guiding role throughout the training process. In the overall objective function, $L_{\mathrm{ppo}}$ ensures policy effectiveness and stability, $L_{\mathrm{ent}}$ enhances policy exploration, $L_{\exp}$ handles specific termination conditions and imposes rule constraints through $\pi_{B}$, while $L_{\mathrm{imi}}$ helps the student model absorb knowledge from the teacher model, ensuring it achieves optimal policy in less time.

### 4.3. Knowledge Distillation-Enhanced Behavior Transformer Algorithm

The proposed KD-BeT algorithm is shown in Algorithm 1. The required inputs include the expert demonstration dataset D, batch sizes $B_{IL}$ and $B_{RL}$, iteration counts for the two learning stages $N_{i}$ and $N_{e}$, as well as learning rates $\alpha_{IL}$ and $\alpha_{RL}$, among other parameters. The entire training process is primarily divided into two stages: Imitation Learning for teacher policy and Reinforcement Learning for student policy with knowledge distillation. In the IL stage, the expert demonstration dataset is initially split into batches of size $B_{IL}$ (lines 1–3). Each batch is then trained iteratively, with the data in each batch being processed through the teacher model to compute the MSE loss function, after which the parameters of the teacher model are updated in a loop (lines 4–11). In the RL stage, the replay buffer R is first initialized, followed by obtaining initial observations from the online environment (lines 12–15). At each time step, the student model outputs the action for the current time step based on the observation–action history, while the teacher model outputs the action for the current time step based on the current observation. The student model’s action is then executed, and the subsequent observation and reward are obtained, storing this information in the replay buffer (lines 16–23). A batch of size $B_{RL}$ is then sampled from the replay buffer to calculate the objective function and update the student model’s parameters θ (lines 24–27). Throughout the training process, the student model learns from the teacher model via knowledge distillation, improving the training efficiency and decision accuracy of the student model.
**Algorithm 1** Knowledge Distillation-Enhanced Behavior Transformer (KD-BeT)**Require:** expert demonstration dataset D, batch size $B_{IL},B_{RL}$, IL iterations $N_{i}$, learning rate $\alpha_{IL},\alpha_{RL}$, RL iterations $N_{e}$, timesteps of episode $N_{t}$, context length *K*, online env
 1:⊳ *Imitation Learning for Teacher Policy* 2:**for** each iteration *i* in $N_{i}$ **do** 3:      Sample trajectories of batch size $B_{IL}$ from expert demonstration {τ}∼D 4:      **for** each τ in {τ} **do** 5:           Predict action ${\hat{a}}_{t}$ by teacher policy ${\hat{a}}_{t}∼\pi_{\theta }^{\mathrm{TE}}\left(\cdot |o_{t}\right)$ 6:           Compute the loss function 7:           $L^{\mathrm{TE}}\left(\pi_{\theta }^{\mathrm{TE}}\right)=E\left[{∥a_{t}^{E}-{\hat{a}}_{t}\left(\pi_{\theta }^{\mathrm{TE}}\right)∥}^{2}\right]$ 8:           update policy parameters $\theta^{TE}$ 9:           $\theta_{k+1}^{\mathrm{TE}}=\theta_{k}^{\mathrm{TE}}-\alpha_{IL}\cdot \nabla L^{\mathrm{TE}}\left(\pi_{\theta }^{\mathrm{TE}}\right)$10:      **end for**11:**end for**12:⊳ *Reinforcement Learning for Student Policy with Knowledge Distillation*13:**for** each iteration *e* in $N_{e}$ **do**14:      Initialize the replay buffer R15:      Get the initial observation $o_{1}$ from online env16:      **for** each timestep *t* in $N_{t}$ **do**17:           Predict action $a_{t}$ by student policy at current timestep $T_{obs}$18:           ${\hat{a}}_{t}∼\pi_{\theta }\left(\cdot |{\left\{o_{t},a_{t}\right\}}_{T_{obs}-K}^{T_{obs}-1}\cup \left\{o_{T_{obs}}\right\}\right)$19:           Predict action $a_{t}^{\mathrm{TE}}$ by teacher policy at current timestep $T_{obs}$20:           ${\hat{a}}_{t}^{\mathrm{TE}}∼\pi_{\theta }^{\mathrm{TE}}\left(\cdot |o_{T_{obs}}\right)$21:           Execute the action and get $s_{t+1},r_{t}$22:           Store the transition $\left(o_{t},{\hat{a}}_{t},{\hat{a}}_{t}^{\mathrm{TE}},r_{t},o_{t+1}\right)$ in R23:      **end for**24:      Sample transitions of batch size $B_{RL}$ from R25:      Compute the objective function and update student policy parameters θ26:      $\theta_{k+1}=\arg\max_{\theta }E_{\tau ∼\pi_{\theta_{k}}}\left[L_{\mathrm{ppo}}+L_{\mathrm{ent}}+L_{\exp}+L_{\mathrm{imi}}\right]$27:**end for**


## 5. Experiment

The experiments were conducted on the CARLA high-fidelity autonomous driving simulator [57] to evaluate the performance of KD-BeT. This section first details the parameters and benchmarks used in the implementation, then evaluates the model’s performance in comparison with current state-of-the-art methods. Finally, a comprehensive ablation study is conducted to validate the model.

### 5.1. Experimental Settings

The experimental evaluation in this study was conducted using CARLA simulator version 0.9.10. During the teacher model training phase, we utilized the Roach framework [56] to build a large-scale expert demonstration dataset, which covers diverse town scenarios and weather conditions, containing over 3 million time steps of high-quality driving data. In the Reinforcement Learning training phase, we implemented an efficient PPO-clip algorithm based on [58]. To significantly improve training efficiency, we adopted a parallel training strategy, deploying six CARLA simulators simultaneously in each training iteration. During model development, we used the PyTorch deep learning framework to build and optimize both Imitation Learning and Reinforcement Learning models. All experiments were conducted on a high-performance computing platform equipped with an NVIDIA GeForce RTX 4090 GPU. In the specific training process, the Imitation Learning training cycle for the teacher model took approximately 5 h, while the Reinforcement Learning training of the student model on six parallel CARLA servers required about 120 h. Notably, in actual inference, the student model achieved an inference latency of only 30 milliseconds on a single RTX 4090 GPU, fully meeting the strict real-time inference requirements of autonomous driving systems. Table 1 lists the detailed hyperparameters for both Imitation Learning and reinforcement learning.

To assess the performance of the trained model, the NoCrash benchmark [33] was used, which is designed to evaluate the driving capability of autonomous systems in a simulated environment. In the training phase of the NoCrash benchmark, the model was trained in Town01. Town01 is a European-style town with single-lane roads and T-junctions, making it well suited to evaluate the model’s performance in basic urban driving scenarios. Specifically, Town01 includes 25 routes with a total driving distance of 17.4 km and 110 traffic lights along the way. The testing phase was conducted in Town02, a scaled-down version of Town01 with additional variations, to evaluate the model’s zero-shot adaptation capabilities. Town02 also includes 25 routes, covering a total distance of 8.9 km with 94 traffic lights. Neither Town01 nor Town02 have stop signs. Additionally, NoCrash defines three traffic densities, Empty, Regular, and Dense, representing increasing levels of traffic density, which serve to evaluate the model’s generalization and adaptability across different traffic conditions.

The primary evaluation metric in the NoCrash benchmark is the success rate, defined as the percentage of test runs in which the autonomous driving system successfully reaches the target location without any collisions. If the vehicle reaches its destination but experiences a collision during the journey, that attempt is not considered successful. Consequently, the success rate serves as a key metric for assessing the model’s performance in a simulated environment, reflecting the system’s safety and reliability.

### 5.2. Comparison with State of the Art

The teacher policy is first trained through IL, then knowledge distillation accelerates the training of the student policy, and the trained model achieves a significant improvement in success rates on the NoCrash benchmark. Table 2 presents a comparison of success rates between KD-BeT and current state-of-the-art methods across different traffic density conditions in both training and testing scenarios. The compared methods include CILRS [33], LBC [35], WOR [44], CADRE [45], GRIAD [46], and RLfOLD [47]. Among them, CILRS and LBC are IL-based methods, while WOR, CADRE, GRIAD, and RLfOLD are RL-based methods.

As shown in Table 2, in the sparse environment of the training scenario, both KD-BeT and RLfOLD achieved a 100% success rate, demonstrating their excellent zero-collision performance in sparse settings. Compared to traditional IL methods like CILRS and LBC, KD-BeT effectively inherits the advantages of the teacher policy through knowledge distillation, significantly improving model performance. In normal- and high-density traffic environments, WOR achieved the best results with success rates of 100% and 96%, respectively, mainly due to its use of model-based RL, which delivers outstanding performance in training scenarios. In comparison, KD-BeT reached success rates of 96% and 93% under the same conditions, slightly lower than WOR and GRIAD but still demonstrating a high degree of success and stability in training scenarios. This demonstrates that KD-BeT achieves comparable performance to state-of-the-art methods in training scenarios by combining the advantages of knowledge distillation and Reinforcement Learning.

In the testing scenario, KD-BeT achieved success rates of 100%, 94%, and 85% in sparse, normal, and high-density environments. Compared to other methods, KD-BeT demonstrates superior generalization capabilities in testing scenarios, primarily due to its unique teacher–student training paradigm and objective function design. Notably, in high-density traffic environments, KD-BeT’s performance advantage becomes more pronounced, showing an approximately 20-percentage-point improvement over WOR and RLfOLD, and a more than 60-percentage-point improvement compared to CILRS and LBC. This result indicates that KD-BeT performs excellently across different traffic densities, particularly maintaining robust performance in high-density environments, highlighting its strong generalization and zero-shot adaptation capabilities in testing scenarios. Additionally, we also conducted tests on the Town05 map to further evaluate the generalization ability of KD-BeT. The decision-making processes for waiting for the preceding vehicle to pass and for waiting at traffic signals are illustrated in Appendix B.

### 5.3. Ablation Study

To evaluate the impact of different components of KD-BeT on model performance, an ablation study was conducted and is presented in this section. Specifically, the influence of different terms in the objective function was analyzed. Ablation experiments were performed on the $L_{\mathrm{ent}}$, $L_{\exp}$, and $L_{\mathrm{imi}}$ terms to assess their effects on model performance.

**Figure 5:**
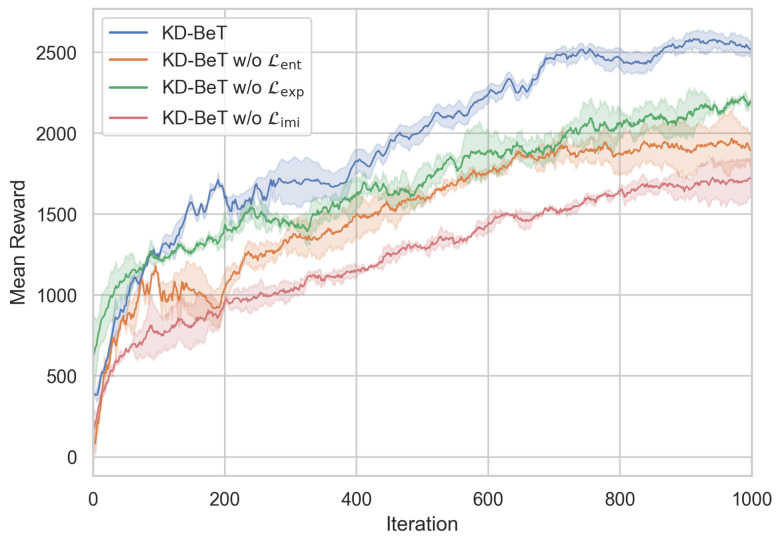
Performance comparison between KD-BeT and ablation of objective function terms during training.

The average reward comparison curve for the RL training process of the student model is shown in Figure 5. As seen in the figure, the KD-BeT method exhibits strong performance during training, with fast convergence and good stability. After removing each of the three terms individually from the objective function, both the convergence speed and asymptotic performance of the model decreased. Removing the $L_{\exp}$ term had the least impact on model performance, while removing the $L_{\mathrm{ent}}$ term led to a more noticeable decrease, indicating that the entropy objective plays an important role in model exploration and stability. The most significant performance drop occurred when the $L_{\mathrm{imi}}$ term was removed, suggesting that knowledge distillation from the teacher policy is crucial for the model’s learning and generalization abilities.

**Figure 6:**
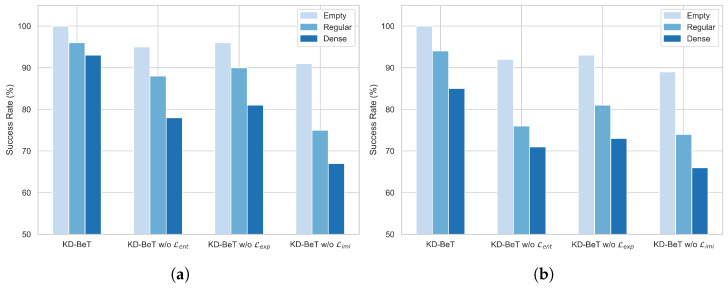
Success rate comparison on the NoCrash benchmark: (**a**) Evaluation on training scenarios. (**b**) Evaluation on testing scenarios.

Additionally, an ablation study was conducted during evaluation and the results are shown in Table 3. Evaluations were conducted from three perspectives: success metrics, collision metrics, and other metrics. The success rate belongs to the success metrics, and the evaluation performance is shown in Figure 6. Besides the success rate, the success metrics include driving score, route completion, and infraction score. Route completion represents the percentage of the route the vehicle actually traversed relative to the total route length, reflecting the model’s ability in navigation and route-following. The infraction score measures the extent of infractions during driving, such as collisions, running red lights, and driving against traffic. A lower IS score indicates more infractions. The driving score, a comprehensive measure of overall driving performance, is calculated as the product of route completion and the infraction score. A high driving score indicates that the vehicle not only completed more of the route successfully but also adhered to traffic rules, reducing infractions and collisions.

Collision metrics include collision with other vehicles, collision with other objects, and red light infractions, indicating the number of incidents for each type. Other metrics include vehicle blockage, representing the number of times the vehicle stopped due to blockages or other reasons.

As shown in Figure 6 and Table 3, in line with training performance, the model performed worst without the $L_{\mathrm{imi}}$ term. Success rate, driving score, route completion, and infraction score were all lower, while collisions with vehicles, objects, red light infractions, and vehicle blockage counts were higher. This underscores the importance of the teacher policy for model performance. Performance decreased the least without $L_{\exp}$ and fell to an intermediate level when $L_{\mathrm{ent}}$ was removed.

The impact of ablating different terms on the model’s training and testing performance leads to the following conclusions: the teacher policy distillation term has the most significant impact on performance improvement. The teacher–student training paradigm plays a key role in enhancing both training efficiency and overall performance. The entropy term also has a substantial impact, primarily in encouraging exploration and preventing premature convergence. Comparatively, the exploration term has the smallest effect on performance, as it mainly functions in specific scenarios—such as imminent collisions, running red lights, encountering stop signs, blockages, or route deviations—where it corrects specific errors and enhances driving safety.

## 6. Conclusions

This paper presents the KD-BeT algorithm, which is based on a “teacher–student” model in autonomous driving. The teacher model is first trained using IL, while the student model learns through knowledge distillation within RL. The Behavior Transformer is employed as the policy network in RL, leveraging observation–action histories to enable sequential decision-making. With the context-learning capabilities of the Transformer, KD-BeT significantly improves decision-making accuracy and demonstrates superior generalization ability in zero-shot scenarios. The KD-BeT framework achieved the best performance on the NoCrash benchmark, showcasing exceptional generalization, zero-shot adaptability, and overall advantages.

This research has achieved significant performance breakthroughs in autonomous driving by innovatively combining IL and RL. However, there are still some challenges in actual deployment. Firstly, in real environments, sensor data quality is often affected by factors such as weather and lighting, leading to noise and uncertainty, which may impact the model’s decision quality. To address this issue, multi-sensor fusion technology can be used to improve data reliability, thereby providing more stable and accurate perception data in various environments. Secondly, model training and inference efficiency is also an important challenge. In terms of training, training on datasets is both time-consuming and prone to overfitting issues. To this end, we will explore more advanced training algorithms, such as meta-learning, implement distributed training based on data parallelism, model parallelism, and simulator parallelism, and adopt data augmentation techniques to accelerate training efficiency. For inference, limited computing resources in vehicle environments place higher demands on real-time decision-making and control. To meet these needs, future work will explore model compression and quantization methods to reduce model size, decrease memory usage, and accelerate inference. Thirdly, although Roach provides high-quality demonstration data as an expert agent for data collection, it relies on privileged information (such as complete road, lane, traffic signal data, etc.) for decision-making, which is difficult to obtain in real environments. Therefore, future work will consider using domain randomization techniques to enhance data diversity, combine real-world data to supplement simulation data, reduce dependence on privileged information, and design more robust learning algorithms to improve model generalization ability. Ultimately, through these measures, we hope to develop more robust and adaptive autonomous driving systems that better handle complex real-world environments.

Looking ahead, we will explore the application prospects of this method in more challenging driving environments, including driving under different weather conditions, high-density traffic scenarios, and complex urban road systems. To address these challenges, we will focus on optimizing model architecture and training strategies to further enhance system performance and generalization capabilities in complex scenarios. In terms of technical innovation, we plan to deeply integrate cutting-edge technologies with the existing framework. The introduction of large language models (LLMs) may bring about a breakthrough in progress—their excellent context understanding and reasoning capabilities will help systems better understand complex traffic scenarios and make more intelligent and reasonable decisions. Meanwhile, we will also explore the potential applications of diffusion models in autonomous driving, leveraging their powerful generative capabilities to improve system decision-making efficiency, safety, and overall performance. Additionally, we will introduce Reinforcement Learning from Human Feedback (RLHF) to enable models to better understand and adapt to human driving behavior characteristics, thereby achieving a more natural and humanized autonomous driving experience. These innovative improvements and optimizations will lay a solid foundation for building safer and more reliable autonomous driving systems.

## Figures and Tables

**Table 1:** Hyperparameters used in the experiments.

Parameter	Value
**Imitation Learning**	
Optimizer	Adam
Learning Rate	5 × 10^−4^
Batch Size	256
Iterations $N_{i}$	50
Teacher Policy Hidden Size	[512, 512, 256, 128]
**Reinforcement Learning**	
Learning Rate	1 × 10^−5^
Minibatch Size	256
Iterations $N_{e}$	2000
Epochs per Iteration	20
time steps per Iteration $N_{t}$	12,288
Context Length *K*	5
Discount Factor γ	0.99
MLP Ratio	2.0
Number of Attention Heads $n_{h}$	4
Number of Blocks $n_{b}$	4
Embedding Dimension	192
GAE Coefficient $\lambda_{\mathrm{GAE}}$	0.9
Clip Range ϵ	0.2
Entropy Coefficient $\lambda_{\mathrm{ent}}$	0.01
Exploration Coefficient $\lambda_{\exp}$	0.05
Distillation Coefficient $\lambda_{\mathrm{imi}}$	0.1
Target KL	0.01
Max Gradient Norm	0.5
Reward Coefficient $\omega_{1},\omega_{2},\omega_{3},\omega_{4},C_{1}$	5, 0.5, 1, 5, 0.1

**Table 2:** Comparison of test results for different methods, focusing on the success rate across three different traffic density conditions.

Method	Source	Success Rate ↑ (%)					
		**Training Scenarios**			**Testing Scenarios**		
		**Empty**	**Regular**	**Dense**	**Empty**	**Regular**	**Dense**
CILRS [33]	CVPR 19	97	83	42	66	56	24
LBC [35]	CoRL 20	89	87	75	36	36	12
WOR [44]	ICCV 21	98	**100**	**96**	78	82	66
CADRE [45]	AAAI 22	95	92	82	78	72	52
GRIAD [46]	Robotics 23	98	98	94	69	63	52
RLfOLD [47]	AAAI 24	**100**	94	90	**100**	86	66
KD-BeT	Ours	**100**	96	93	**100**	**94**	**85**

**Table 3:** Driving performance and infraction analysis of KD-BeT on the NoCrash benchmark with dense traffic in the testing scenarios. Results are reported as the mean and standard deviation over three evaluation speeds.

Method	Success Metrics (↑)				Collision Metrics (↓)			Other Metrics (↓)
	**Success** **Rate**	**Driving ** ** Score**	**Route ** ** Compl.**	**Infrac. ** ** Score**	**Collision ** ** Others**	**Collision ** ** Vehicle**	**Red Light ** ** Infraction**	**Vehicle ** ** Blocked**
KD-BeT w/o $L_{\mathrm{ent}}$	71±3	75±6	91±4	79±5	0.57±0.39	0.87±0.35	0.75±0.43	1.52±0.45
KD-BeT w/o $L_{\exp}$	73±5	78±4	94±2	81±3	0±0	0.67±0.49	0.56±0.31	0.92±0.19
KD-BeT w/o $L_{\mathrm{imi}}$	68±8	69±9	86±2	78±2	0.86±0.54	1.75±0.86	1.59±0.23	2.16±1.65
KD-BeT	85±3	87±4	100±0	87±4	0±0	0.32±0.25	0.24±0.17	0±0

**Figure A1:**
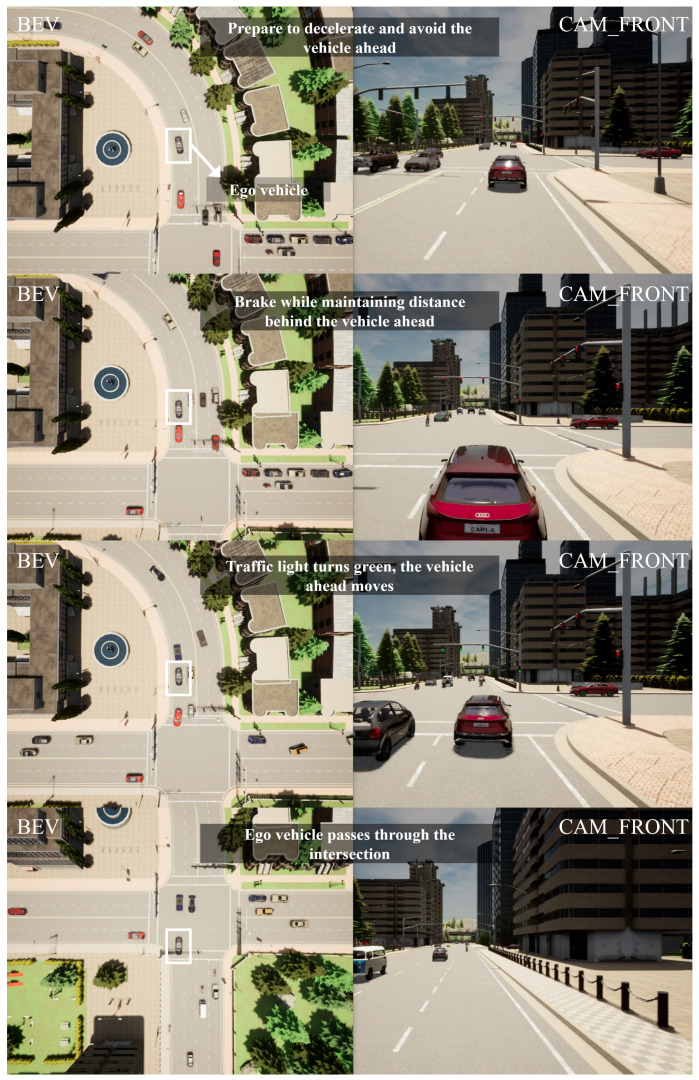
Illustration of ego vehicle’s decision-making process: waiting for the vehicle ahead and passing through the intersection.

**Figure A2:**
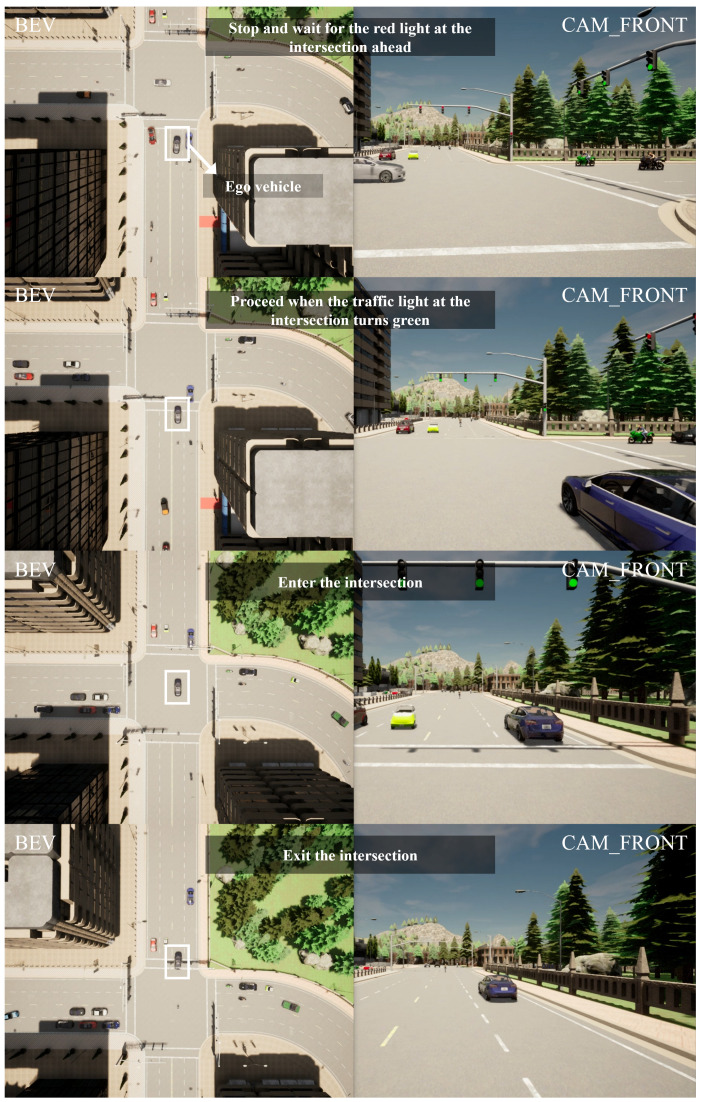
Illustration of ego vehicle’s decision-making process: waiting and passing through the intersection with a traffic light.

## Data Availability

The data presented in this study are available upon request from the corresponding author.

## Appendix A

In this section, the structure and implementation of the Behavior Transformer are introduced. The Transformer model is a deep learning model based on a self-attention mechanism, which is widely used in natural language processing, computer vision, and other fields. In autonomous driving decision-making tasks, the Transformer model captures temporal dependencies in input sequences through the self-attention mechanism, realizes a deep feature extraction of input sequences, and thus achieves more accurate decision prediction. The following content will introduce the core structure and implementation principles of the Transformer model in detail.

In the self-attention mechanism, the model learns contextual information at each time step by computing correlations between tokens, enabling more accurate decision predictions. Self-attention, the core of the Transformer, captures temporal dependencies in the input sequence by assigning different weights to each token. The key idea behind self-attention is using query (*Q*), key (*K*), and value (*V*) representations to compute relationships between tokens. The input sequence $F^{in}\in R^{M\times D_{f}}$ represents *M* tokens, each represented by a feature vector of dimension $D_{f}$. Through linear transformations, the model computes query, key, and value representations as follows:

(A1) $$\begin{array}{c} Q=F^{in}W^{q},\;K=F^{in}W^{k},\;V=F^{in}W^{v} \end{array}$$

where $W^{q}$, $W^{k}$, and $W^{v}$ are learnable weight matrices in the model. The core of the self-attention mechanism lies in computing correlations between tokens through scaled dot products of queries and keys. These correlation weights are then normalized through a softmax function and used to weight the value tokens to generate output features. The specific formula is

(A2) $$\begin{array}{c} \mathrm{Attn}\left(Q,K,V\right)=\mathrm{softmax}\left(\frac{QK^{T}}{\sqrt{d_{k}}}\right)V \end{array}$$

where $d_{k}$ is the dimension of the keys. This allows the model to automatically identify the most relevant observations and actions in the input sequence for current decisions. To enhance the model’s expressiveness, the Transformer also employs a multi-head attention mechanism. This mechanism computes multiple independent attention heads in parallel, learning feature representations from different subspaces. Each attention head independently computes attention, and the results are then concatenated and transformed through a linear layer to generate the final output. The multi-head attention computation formula is

(A3) $$\begin{array}{c} \mathrm{MultiHead}\left(Q,K,V\right)=\mathrm{concat}\left({\mathrm{head}}_{1},\cdots ,{\mathrm{head}}_{h}\right)W^{O} \end{array}$$

where each attention head ${\mathrm{head}}_{i}$ is computed similarly to single-head attention:

(A4) $$\begin{array}{c} {\mathrm{head}}_{i}=\mathrm{Attn}\left(Q_{i},K_{i},V_{i}\right) \end{array}$$

where $W^{O}$ is the output weight matrix for the multi-head attention mechanism. In addition to self-attention, the Transformer model introduces positional encoding to capture temporal information in the input sequence, enabling it to perceive the order of the input sequence. Since the Transformer model is inherently sequence-agnostic, we added sinusoidal positional encoding to each input token, allowing the model to learn relative position information in the input sequence. In this way, the model can attend not only to important observations in the input sequence but also to their temporal structure.

To handle sequential data, we further adopted a causal Transformer. The causal Transformer restricts each token to only attend to previous tokens, ensuring that the model’s output does not depend on future information, thus maintaining temporal consistency. This is crucial for decision-making tasks in autonomous driving, as vehicle decisions can only be based on past observations and cannot “peek” into future situations. The causal Transformer effectively meets this requirement.

Finally, after computing self-attention, the Transformer applies an MLP to perform nonlinear transformations on the generated features. Specifically, the MLP processes each attention result and adds it to the input features $F^{in}$ to generate the final output features $F^{out}$:

(A5) $$\begin{array}{c} F^{out}=\mathrm{MLP}\left(\mathrm{Attn}\left(Q,K,V\right)\right)+F^{in} \end{array}$$

Through this structure, the Transformer can perform deep feature extraction on input sequences and generate corresponding action decisions based on these features. Due to the Transformer’s powerful self-attention and multi-head attention mechanisms, it excels at handling complex temporal dependencies, making it particularly suitable for autonomous driving tasks in dynamic environments.

## Appendix B

In this section, we provide a detailed demonstration of the intelligent decision-making process of the ego vehicle in complex traffic scenarios. Specifically, we focus on analyzing two typical scenarios: first, the ego vehicle’s decision behavior of waiting for the vehicle ahead to pass through an intersection, as shown in Figure A1; and second, the ego vehicle’s waiting decision process at traffic signals, as shown in Figure A2. These scenarios fully demonstrate our system’s decision-making capabilities and safety awareness in real traffic environments.

Figure A1 illustrates the decision-making process of the ego vehicle, i.e., waiting for the vehicle ahead to pass through the intersection. The process consists of four main stages: First, detecting a vehicle ahead and preparing to slow down to yield. Second, maintaining a safe following distance and braking behind the front vehicle. Third, the traffic light turns green and the front vehicle proceeds. Finally, with no vehicle ahead and a green light, the ego vehicle advances and passes through the intersection.

Figure A2 illustrates the decision-making process of the ego vehicle, i.e., waiting for the traffic light to turn green. The process consists of four main stages: First, stopping at the red light at the intersection ahead. Second, proceeding when the traffic light turns green. Third, entering the intersection. Finally, exiting the intersection.
